# Mixed genetic background better recapitulates developmental and psychiatric phenotypes and heterogeneity than inbred C57BL/6J mice

**DOI:** 10.1038/s41598-025-26215-6

**Published:** 2025-11-07

**Authors:** Ana Dudas, Ana Novak, Caroline Gora, Emmanuel Pecnard, Nicolas Azzopardi, Séverine Morisset-Lopez, Lucie P. Pellissier

**Affiliations:** 1 INRAE, CNRS, Université de Tours, PRC, Nouzilly, 37380 France; 2 Center for Molecular Biophysics-CNRS UPR 4301, Rue Charles Sadron, Orléans, France

**Keywords:** Inbred mouse strain, Mixed genetic background, Neurodevelopmental and psychiatric conditions, Social interaction, Translational research, Genetics, Neuroscience

## Abstract

Preclinical models of neurodevelopmental and psychiatric conditions often rely on inbred mouse strains like C57BL/6J (B6), which exhibit limited genetic and behavioral variability. This limitation hampers the modeling of phenotypic heterogeneity, characteristic of these conditions. Recent efforts have explored the use of multiple genetically diverse hybrid strains to address this. In this study, we examined whether one single mixed genetic mouse background, C57BL/6J;129S2/SvPas (B6;129), could simultaneously recapitulate behavioral variability and display improved phenotypes relevant to neurodevelopmental and psychiatric conditions. Compared to the inbred B6 strain, mixed B6;129 mice displayed enhanced sociability and self-grooming, two key discriminating parameters between the two mouse backgrounds and core features of such conditions, alongside a broader spectrum of individual behavioral variability. Although overall behavioral variability was comparable across backgrounds, B6;129 mice were less susceptible to the effects of chronic social isolation than their B6 counterparts. Together, these findings support that the B6;129 mixed background offers a more representative model of individual variability and behavioral traits associated with neurodevelopmental and psychiatric conditions, thereby enhancing its translational relevance in preclinical studies.

**Supplementary Information:**

The online version contains supplementary material available at 10.1038/s41598-025-26215-6.

## Introduction

Neurodevelopmental and psychiatric conditions, such as schizophrenia or autism spectrum disorder (ASD), are defined by the Diagnostic and Statistical Manual of Mental Disorders^1^, based on the behavioral criteria, with deficits in social interaction being a common feature. These conditions are widely heterogeneous across individuals, driven by complex interactions between genetic predispositions and environmental factors, which together shape neural circuitry and behavioral outcomes^2–4^.

Over the past two decades, mice have served as valuable models for studying those conditions and testing new treatments, as they can exhibit both impaired social behaviors and co-occurring or negative features^5^. Inbred strains, created by mating brother-sister pairs over at least 20 generations to achieve ~ 99% homozygosity, are widely used in neurological research to examine genetic and environmental mechanisms and the effect of potential treatments. Among these, the C57BL/6 (B6) is the most commonly used strain due to its reduced genetic variability and well-characterized genome^6^. B6 mice display average sociability relative to other inbred strains^7,8^. Several inbred strains have even been identified as mouse models of ASD. Notably, BTBR T^+^ tf/J mice exhibit profound social and communication impairments, as well as increased repetitive and stereotyped behaviors compared to B6 mice^8–12^. In contrast, FVB/NJ mice show high sociability, often serving as a control to low-sociability strains in experimental assays^7,8,12^. The 129 Sv (129) strains, commonly used for generating transgenic models, display sociability levels similar to those of B6 mice, although with greater variability, partly mirroring the diversity observed in human conditions^8,13^.

While inbred mice are valuable for studying the effects of single-gene deletions or environmental factors independently, findings derived from these homogeneous backgrounds may not generalize to genetically heterogeneous conditions^14–16^. To address this limitation, genetically diverse mouse populations have been developed to better model complex gene–environment interactions that influence behavioral traits, which are particularly relevant to neurodevelopmental and psychiatric conditions. Outbred strains (e.g., Swiss, CD1) are maintained by random mating in large colonies to preserve genetic diversity. Additionally, the BXD or Collaborative Cross (CC) derived from controlled crossing of inbred strains and/or wild-derived mice and maintained as inbred mouse lines, offer genetic diversity throughout all the lines with fully sequenced genomes^17–20^. Notably, wild-derived mice often exhibit behavioral traits distinct from lab inbred mice; for instance, female wild-derived mice maintain a social hierarchy lost in laboratory strains^21^. A recent study revealed that crossing B6 heterozygous females for the high-confidence ASD risk gene (*Cdh8*^*+/−*^) with 27 reference CC males, used to model human genetic heterogeneity, strongly influences behavioral phenotypes^22^.

F1 hybrids, the direct offspring of two different inbred strains strictly heterozygous at all loci, generally exhibit enhanced physiological robustness compared to inbred lines^23^. However, behavioral studies in hybrid genetically homogeneous mice have focused on memory and anxious-like behaviors^6,23^. In contrast, mixed background mice refer to F2 or later-generation crosses of two inbred strains without intentional genetical selection. They carry a unique, heterogeneous combination of parental alleles. These mixed background mice may better model human genetic diversity, particularly in neurodevelopmental and psychiatric conditions that are influenced by multiple genetic and environmental risk factors^24,25^. Additionally, diversity outbred (DO) mice are a genetically diverse population created by breeding multiple inbred mouse strains together, including B6 and 129 mice, and then systematically outbreeding them^26,27^. Although it has been long debated whether inbred mice are superior to outbred lines^28–31^, in this study, we used mixed background mice and compared them with the most commonly used B6 inbred strain to assess neuro developmental and psychiatric-related behaviors. We compared behavioral phenotypes relevant to these conditions between mixed C57BL/6J;129S2/SvPas (B6;129) background and B6 inbred mice. We also evaluated behavioral variability and explored how an environmental model manifests across these genetic backgrounds.

## Results

### Mixed background mice exhibit enhanced sociability

The B6 is the most commonly used inbred strain in neuroscience research^6^. We investigated whether introducing genetic diversity by mixing B6 with the 129 inbred strain, known for comparable sociability but greater behavioral variability^8,13^, would alter sociability. Therefore, to assess social interaction and its potential variability, we compared inbred B6 mice with mixed background B6;129 mice using standardized social behavior tests^32,33^, including interactions with either a familiar (cage mate) or an unfamiliar conspecific.

**Fig. 1:**
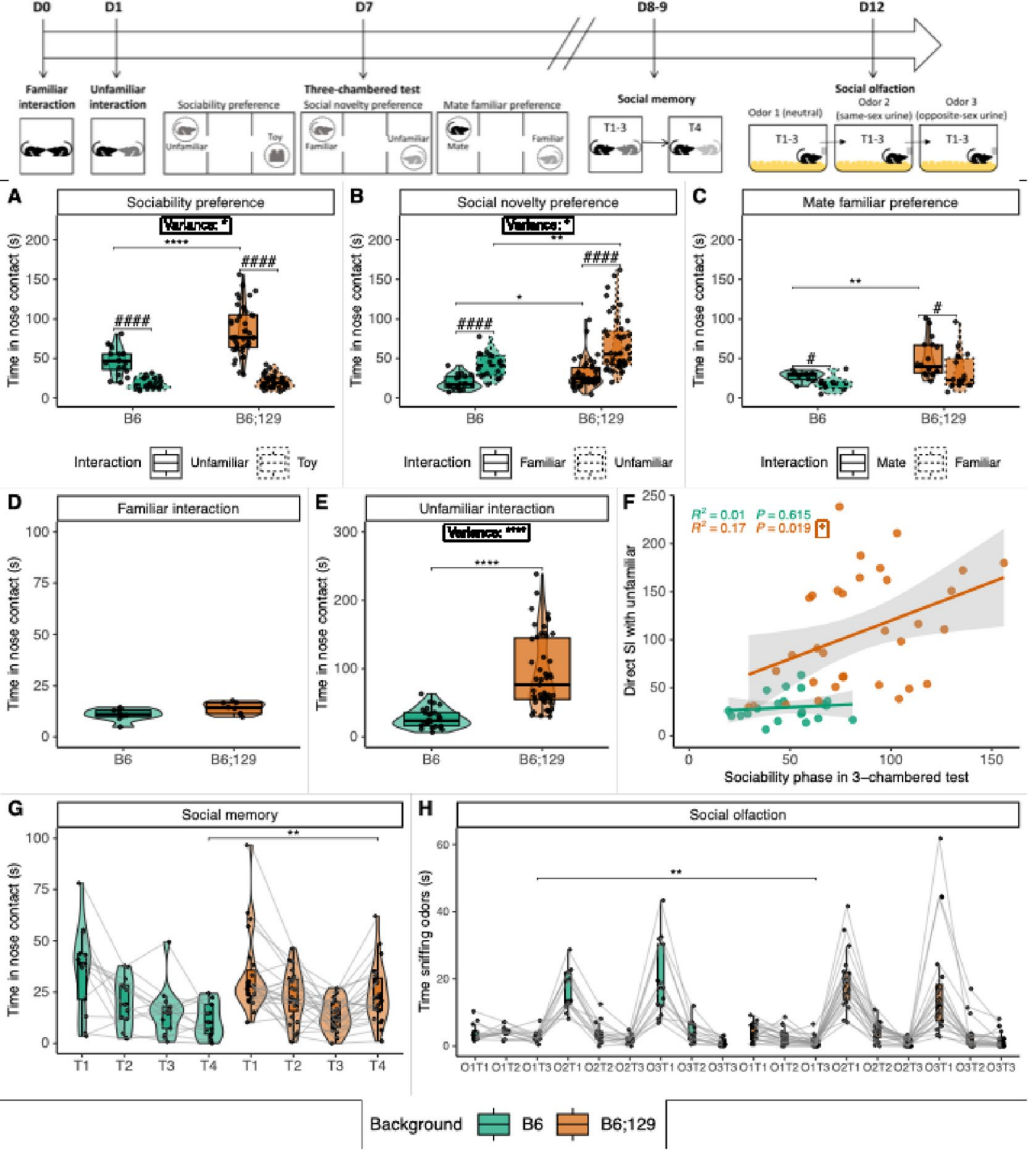
Mixed B6;129 mice exhibit enhanced sociability and variability with unfamiliar mice. Experimental timeline of behavior assays. In the 10 min three phases of the three-chambered test, B6;129 mice (orange) showed enhanced sociability (unfamiliar WT mouse 1 vs. object; (**A**)), social novelty preference (familiar WT mouse 1 vs. novel unfamiliar WT mouse 2; (**B**)) and familiar cage mate (familiar cage mates vs. familiar WT mouse 2; (**C**)) preference compared to B6 mice (green). In the 10 min reciprocal social interaction, B6;129 mice showed enhanced social interaction with a background-, sex- and age-matched familiar cage mate  (**D**) but not unfamiliar mouse (**E**) compared to B6 mice. Nose contact with an unfamiliar mouse in the reciprocal social interaction was positively correlated with those with an unfamiliar mouse in the sociability phase of the three-chambered test (**F**). In the 5 min social memory test (**G**), both B6;129 and B6 mice exhibited similar social recognition and habituation to the WT interactors over the four trials. In the odor habituation-dishabituation assay (**H**), B6;129 and B6 mice displayed similar neutral (O1, blossom flower) and social odor (O2 sex-matched or O3 opposite sex WT urines) recognition. Data are presented as individual data, mean ± sd (statistics, n and sex ratio in Table S1). Groups were compared by Kruskal-Wallis tests followed by Dunn post hoc tests, with stars indicating background effect and hash chamber effect (P = adjusted p-value), while inter-individual variability was compared by Levene’s variance test. * or # *p* < 0.05; ** or ## *p* < 0.01; *** or ### *p* < 0.001; **** or #### *p* < 0.0001.

Strikingly, although both backgrounds were sociable, B6;129 mice showed increased social interaction, particularly with unfamiliar mice, compared to B6 mice in the sociability and social novelty phases of the three-chambered test, as well as in the reciprocal interaction assay (Fig. 1A–E, Table S1). Notably, the higher inter-individual variability observed in B6;129 mice was specific to unfamiliar interactions, as no differences were found in familiar interactions. Moreover, in B6;129 mice, interactions with unfamiliar conspecifics were positively correlated across tests, suggesting stable social behavior traits across tasks and days (Fig. 1F). No significant sex differences were detected across backgrounds (Figure S1, Table S1).

To exclude the possibility that differences in social interaction stemmed from altered social memory or olfactory function, we assessed the performance of both backgrounds using neutral and social odors, as well as subsequent exposure to unfamiliar and familiar mice, in established habituation/dishabituation tests^34,35^. B6 and B6;129 mice performed comparably, with the exception of habituation during the third exposure to the neutral O1T3 odor (Fig. 1G-H). Interaction with unfamiliar animals in trial 4 was also enhanced in B6;129 mice in this test. These results indicate that both strains exhibit relatively similar social memory and olfactory processing.

**Fig. 2:**
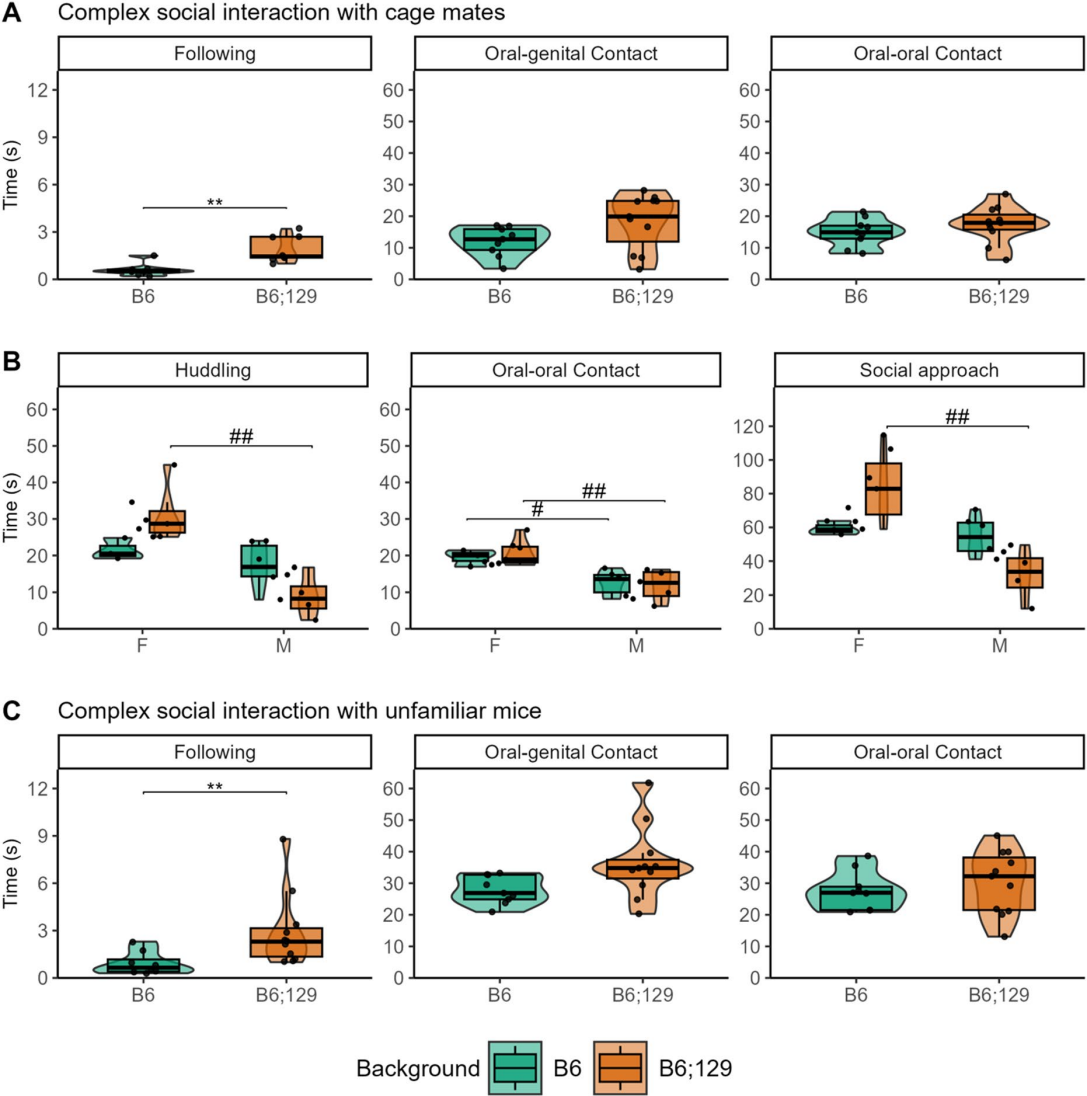
Mixed B6;129 mice show improved social interest and sex differences in the Live Mouse Tracker. In the 10 min complex social interaction in the Live Mouse Tracker, B6 mice (green) and B6;129 mice (orange) displayed increased time following familiar cage mates (**A**,**B**) and in the subsequent trial with a mix of 2 unfamiliar B6 mice and 2 unfamiliar B6;129 mice while no differences in nose contacts were observed (**C**). Sex differences were identified when interacting with familiar cage mates (**B**), with females showing increased time huddling and in social approach compared to males in the B6;129 background, as well as nose-to-nose contacts in both backgrounds. Data are presented as individual data, mean ± sd (statistics, n and sex ratio in Table S2). Groups were compared by Kruskal-Wallis tests followed by Dunn post hoc tests, with stars indicating background effect and hash sex effect (P = adjusted p-value), while inter-individual variability was compared by Levene’s variance test. * or # *p* < 0.05; ** or ## *p* < 0.01; *** or ### *p* < 0.001.

To further explore social interaction in a more complex and dynamic setting, we used the Live Mouse Tracker (LMT) system^36^ to expose B6 and B6;129 mice to either familiar cage mates or a mixed group of four unfamiliar B6 and B6;129 mice over consecutive days (Fig. 2, Table S2). B6;129 mice displayed increased time spent following both familiar and unfamiliar mice, compared to B6 mice (Fig. 2A,C). This increased behavior was not accompanied by elevated oral–anogenital sniffing, a behavior often linked to aversive or aggressive social interactions, particularly in males^37,38^, suggesting that the enhanced following behavior in B6;129 mice reflects increased social interest rather than aggression. Sex-specific differences emerged more clearly in the B6;129 background (Figs. 2B, S2, Table S2). Female B6;129 mice exhibited increased huddling and social approach behaviors toward familiar animals. For nose-to-nose contacts, sex effect was observed in both backgrounds, but was more pronounced in B6;129 mice, indicating that the mixed background enhanced the detection of sex-dependent behavioral differences.

In conclusion, mixed B6;129 background mice display enhanced social interaction toward unfamiliar conspecifics, accompanied by increased variability and sensitivity to sex differences.

### Mixed background mice display increased self-grooming and reduced anxious-like behaviors

**Fig. 3:**
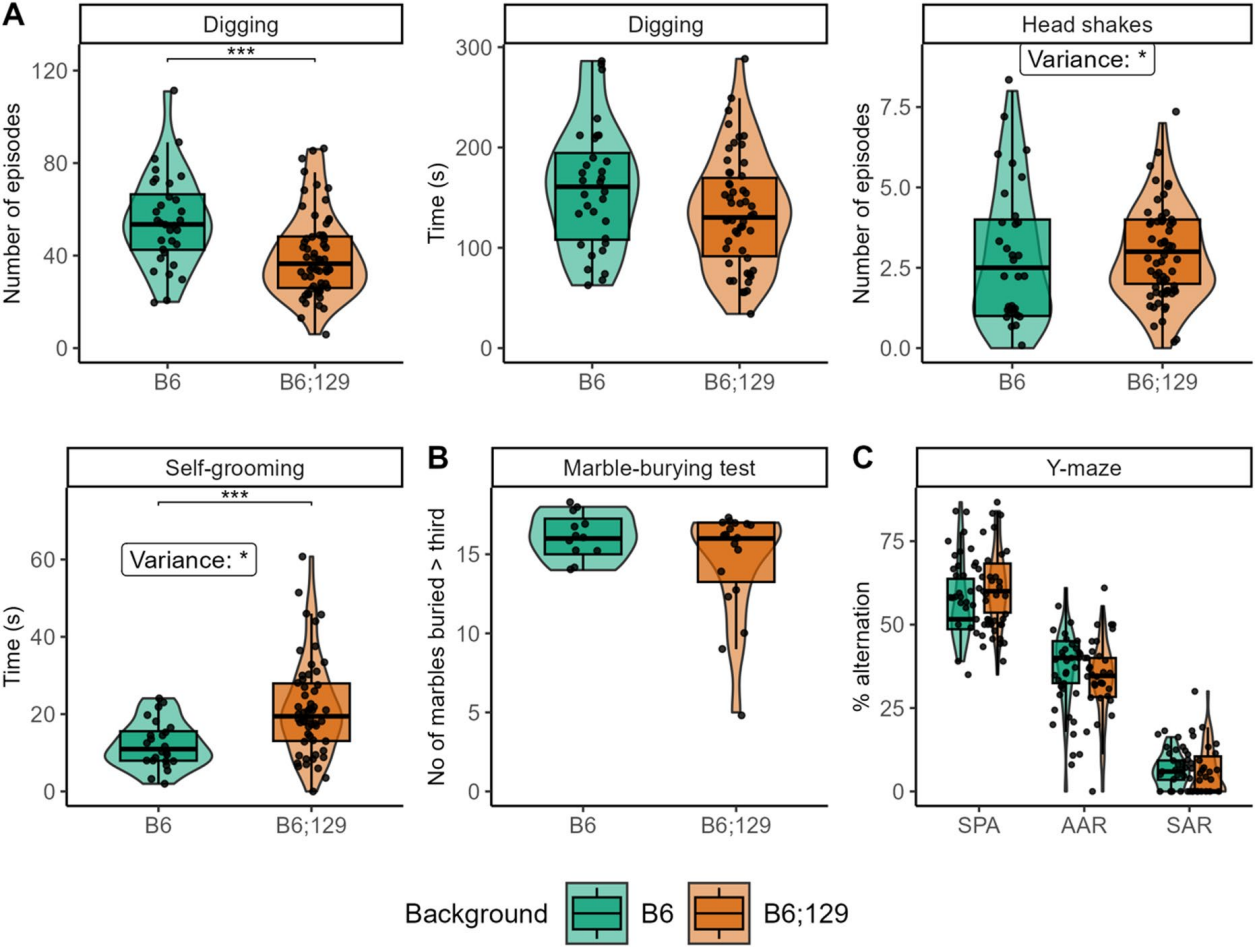
Mixed B6;129 background mice display increased self-grooming. In the 10 min motor stereotypies (**A**), B6 mice (green) displayed enhanced number of digging episodes, and no difference in the time spent digging or head shake episodes, while B6;129 mice (orange) showed increased time in self-grooming. No difference in the marble burying test (**B**) or in the Y maze alternation pattern (**C**) was observed between B6 mice and B6;129 mice. Data are presented as individual data, mean ± sd (statistics, n and sex ratio in Table S1). Groups were compared by Kruskal-Wallis tests followed by Dunn post hoc tests, with stars indicating background effect (P = adjusted p-value), while inter-individual variability was compared by Levene’s variance test. * *p* < 0.05; ** *p* < 0.01; *** *p* < 0.001.

**Fig. 4:**
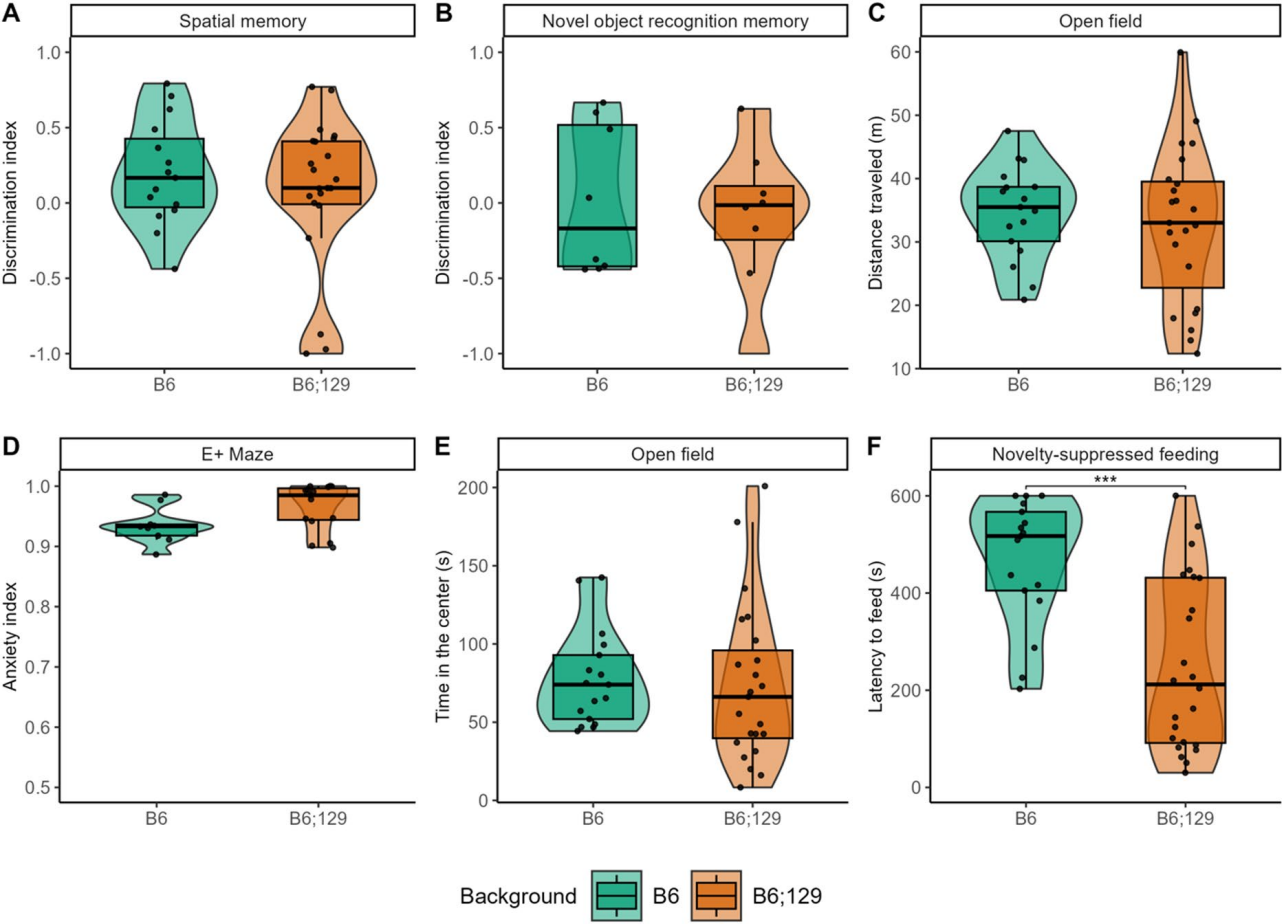
Inbred B6 mice display increased anxious-like behaviors. In the spatial object location (**A**) and novel object (**B**) recognition memory tests, B6 mice (green) and B6;129 mice (orange) performed similarly, as well travelled the same distance in the open field (**C**) for 10 min. No difference in anxious-like behavior was observed in the elevated plus (E+) maze (**D**) or time in the center in the open field arena (**E**) for both backgrounds, while B6 mice showed increased latency to feed in the novelty-suppressed feeding tests (**F**), compared to B6;129 mice. Data are presented as individual data, mean ± sd (statistics, n and sex ratio in Table S1). Groups were compared by Kruskal-Wallis tests followed by Dunn post hoc tests, with stars indicating background effect (P = adjusted p-value), while inter-individual variability was compared by Levene’s variance test. * *p* < 0.05; ** *p* < 0.01; *** *p* < 0.001.

We next assessed whether genetic background influenced stereotyped, compulsive, or restrained behaviors, as well as anxious-like behavior, cognitive flexibility, and locomotion in established tests^33,39,40^ (Figs. 3 and 4, S3–S4, Table S1).

B6;129 mice exhibited increased time in self-grooming, but no differences in the number of head shakes (repetitive and stereotyped behaviors, respectively), while both variances were significantly higher compared to B6 mice (Fig. 3A). In contrast, B6 mice displayed an increased number of digging episodes (but not increased digging time), indicative of natural foraging or heightened anxious-like behaviors^41^. These behavioral effects were primarily driven by male mice (Figure **S3**). However, no differences were observed in marble burying (obsessive-compulsive–like behavior) and spatial Y-maze spontaneous alternation (restrained behavior and cognitive flexibility) between backgrounds (Fig. 3B-C), suggesting that the increased digging in B6 mice likely reflects elevated anxious-like behavior rather than compulsivity.

Regarding other behaviors, no significant differences were found between backgrounds in spatial memory, novel object recognition, or locomotor activity (Fig. 4A-E, Table S1). However, anxious-like behavior was significantly higher in B6 mice compared to B6;129 in the novelty-suppressed feeding test, mainly in females, whereas no differences were observed in the elevated plus maze or in the time spent in the center of the open field (Figs. 4D,F, S4), supporting the anxiety-related behaviors detected via digging behavior. No other significant sex differences were detected across these tests (Figures S3–S4, Table S1).

In conclusion, B6;129 mice displayed increased self-grooming and reduced anxious-like behaviors, compared to B6 mice. Unlike social interaction parameters, behavioral variability in the mixed background was less pronounced for these behaviors.

### Sociability and motor stereotypies are key discriminating parameters between the two backgrounds

**Fig. 5:**
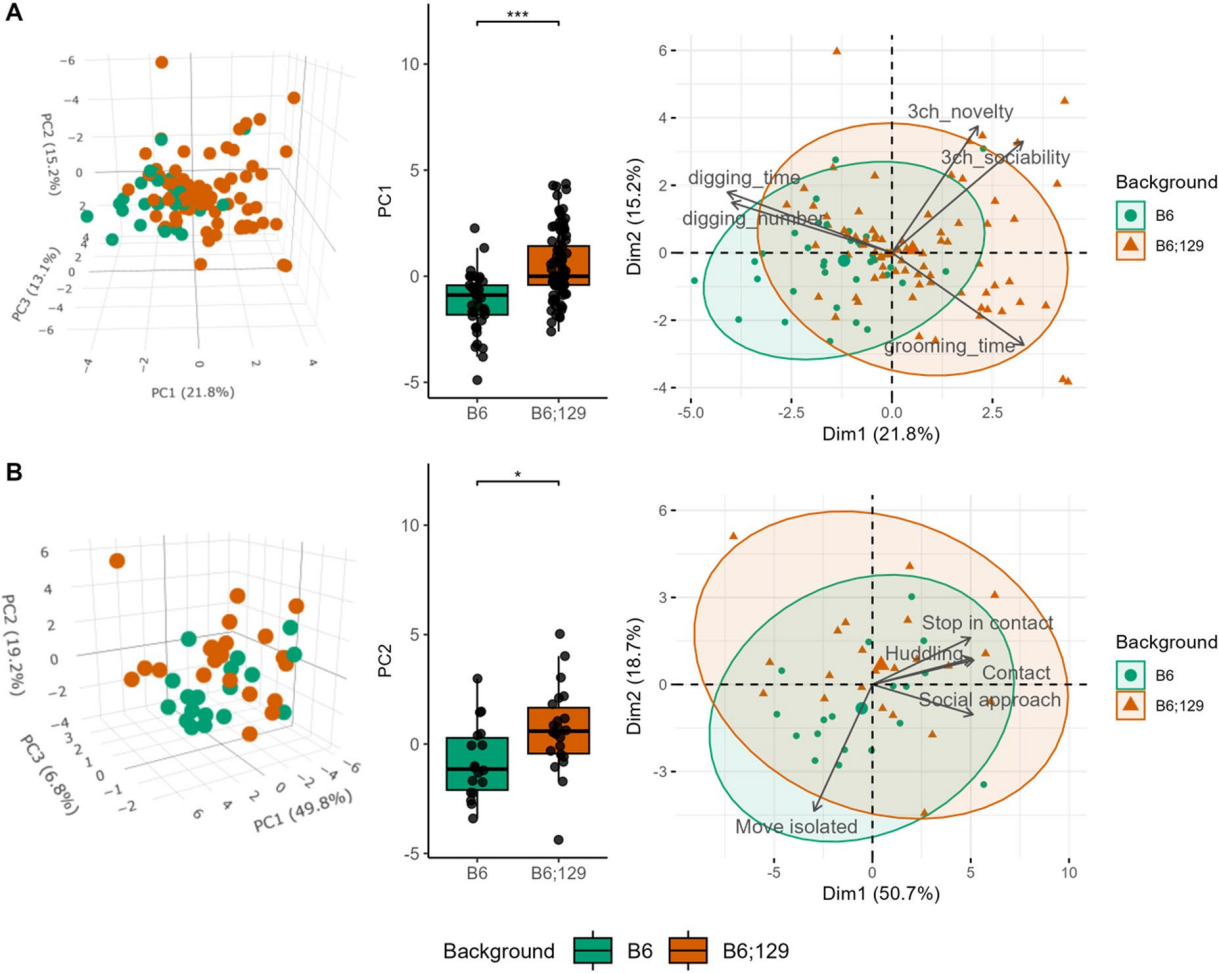
Sociability and motor stereotypies discriminate between backgrounds. Based on the behavioral phenotypes of B6 mice (green) and B6;129 mice (orange) in the reciprocal social interaction, three-chambered, motor stereotypies, Y-maze, open field, and novelty-suppressed feeding tests (**A**), principal component analysis revealed higher variability in B6;129 background in the first principal component (left), confirmed by a significant increase of the scores for the first principal component in B6;129 compared to B6 background (center). The most contributing behavioral parameters to this variability in B6;129 background were sociability and stereotyped behavior parameters (right). Based on the behavioral phenotypes of B6 (green) and B6;129 mice (orange) in the Live Mouse Tracker interacting with cage mates or unfamiliar age- and sex-matched B6 and B6;129 mice (**B**), the principal component analysis revealed increased variability of the B6;129 mice in the second principal component (left), evidenced by an increase in the scores for the second component (center). The most discriminating parameters contributing to this variability in the B6;129 background were social interaction parameters (right). Data are presented as individual data, mean ± sd (Tables S1-2). Groups were compared by the one-way ANOVA followed by Tukey’s posthoc tests, with stars indicating the background effect. *p < 0.05; **p < 0.01; ***p < 0.001.

To identify the key parameters that robustly discriminated between the two backgrounds, we conducted a principal component analysis (PCA) using all behavioral parameters (Fig. 5, Tables S1-2).

Only PC1 (Dim1) significantly differed between B6 and B6;129 mice and was driven primarily by enhanced sociability and social novelty in the three-chambered test and self-grooming behavior in B6;129 mice, as well as increased digging behavior in B6 mice (Fig. 5A). Although less pronounced than in classical tests, PCA analysis applied to the LMT dataset revealed that the top five discriminating factors were social parameters (Fig. 5B). PC2 significantly distinguished the backgrounds, with B6 mice characterized by increased isolation, and B6;129 mice by a greater number of contacts. The results were similar in both males and females (Figure S5).

In conclusion, these analyses demonstrate that sociability and motor stereotypies, both core ASD-like traits, are the key discriminating behavioral parameters between the inbred and mixed genetic backgrounds.

### Mixed background is less sensitive to chronic social isolation, a mouse model of neurodevelopmental and psychiatric conditions

**Fig. 6:**
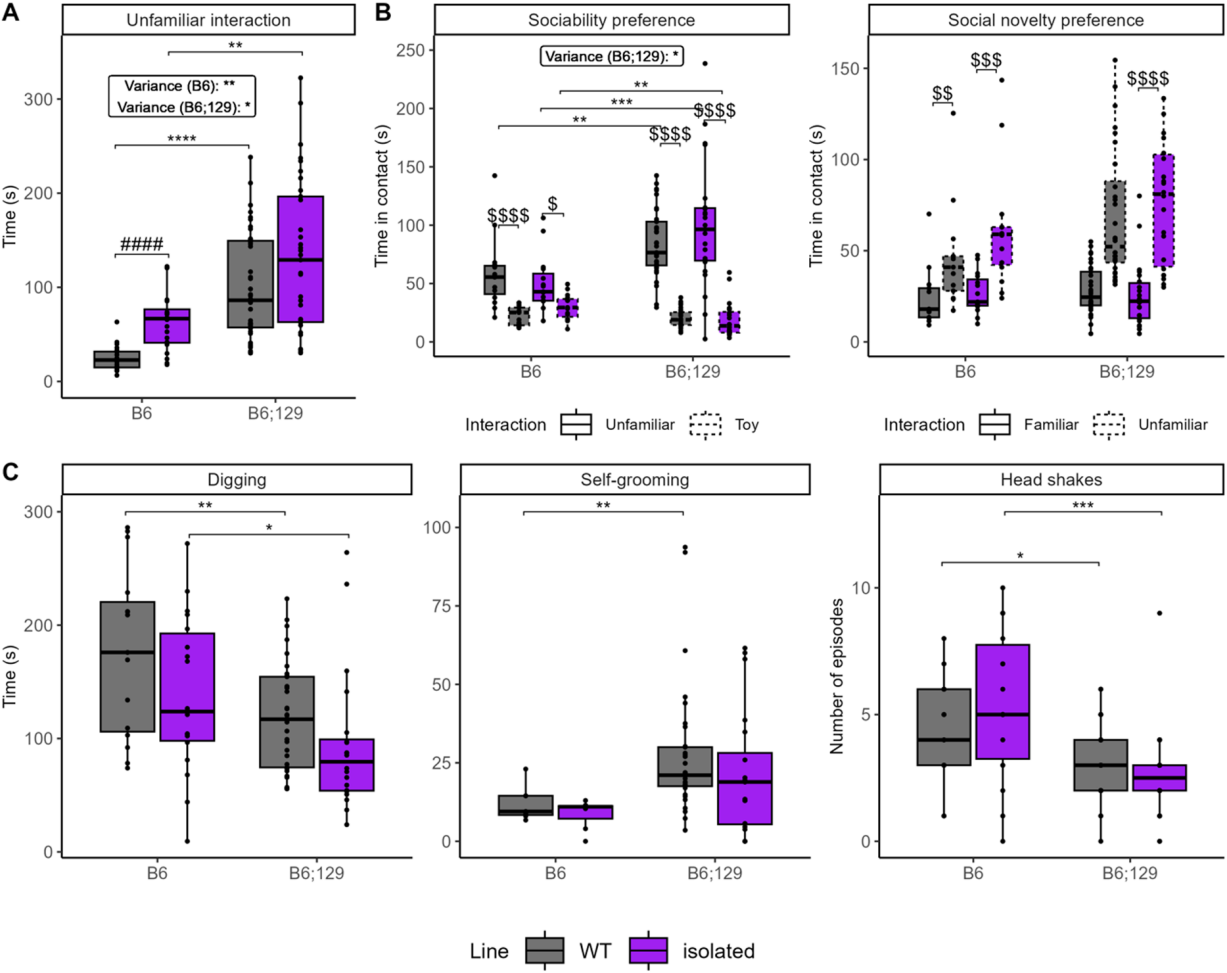
Mixed B6;129 background enhances sociability and self-grooming in WT, but not in chronically isolated mice. In the reciprocal social interaction test, chronically isolated mice (purple) revealed significant differences compared to WT controls (gray) only in the B6 background, while chronic isolation increased variance in both backgrounds. Social interaction was significantly increased in both WT control and isolated mice from the B6;129 compared to B6 background (**A**). In the sociability phase of the three-chambered test, sociability preference was observed for both chronically isolated mice and WT control, while this preference was significantly higher in B6;129 background with a higher variance (**B**). In the motor stereotypy test, there was no difference in the time spent digging or self-grooming between WT and chronically isolated mice, while both WT and isolated mice in B6 background showed increased time spent digging and number of head shakes, while increase in time spent self-grooming was observed in B6;129 WT mice compared to B6 conspecifics (**C**). Data are presented as individual data, mean ± sd (statistics, n and sex ratio in Table S3). Groups were compared by Kruskal-Wallis tests followed by Dunn post hoc tests, with stars indicating background effect, hash housing effect, and dollar chamber effect (P = adjusted p-value), while the inter-individual variability was compared by Levene’s variance test. *, # or $ *p* < 0.05; **, ## or $$ *p* < 0.01; ***, ### or $$$ *p* < 0.001; ****, #### or $$$$ *p* < 0.0001.

To assess whether the mixed B6;129 background better models behavioral heterogeneity in a mouse model of ASD and schizophrenia^42–45^, or whether findings are specific to the inbred B6 background, we compared the effects of four weeks post-weaning chronic social isolation in mice (isolated) in both genetic backgrounds (Fig. 6, Table S3).

B6;129 wild-type mice raised in groups (WT) and isolated mice showed enhanced social interaction with an unfamiliar conspecific across multiple social assays (Fig. 6A,B) and displayed greater inter-individual variability compared to B6 WT mice (Table S3), confirming our previous baseline observations between backgrounds. Chronic social isolation increased social interaction with an unfamiliar, isolated conspecific in the reciprocal interaction test (Fig. 6A,B). This effect was significant only in the B6 background, but did not change social interest in the three-chamber test when interacting with an unfamiliar WT mouse. Interestingly, although sociability was more variable among isolated B6;129 mice compared to their respective WT, isolated B6;129 mice showed a stronger preference for the mouse over the object than isolated B6 mice. This suggests that social interaction in the B6;129 background is less sensitive to the effects of chronic social isolation.

WT B6;129 mice also displayed increased self-grooming compared to WT B6 mice (Fig. 6C), confirming our results in previous cohorts. However, this difference was not observed in isolated animals. Furthermore, both B6;129 WT and isolated mice spent less time digging and displayed fewer head shakes compared to B6 mice. Although chronic isolation was not expected to affect head shakes, a proxy of stereotyped behavior^43,44^, it amplified the behavioral differences between the two genetic backgrounds.

In conclusion, while both backgrounds displayed greater behavioral heterogeneity following chronic social isolation, the mixed B6;129 genetic background appeared less sensitive to its effects.

## Discussion

The main novelty of this study is that, although more variable as expected from their genetically heterogeneous background, mice raised in the mixed B6;129 background exhibited a robust and replicable increase in social interactions with unfamiliar conspecifics compared to those maintained in the inbred B6 background. Although not tested here, and a limitation of this study, the 129 line has been reported to display sociability levels comparable to B6 mice^6,8,13^. Therefore, the enhanced sociability and broader inter-individual variability observed in the B6;129 mixed line likely result from increased genetic diversity rather than from one parental strain dominating the phenotype. This increased social interest may reflect enhanced social motivation, which could be further evaluated in an operant behavior test^46^. Although less pronounced than in the mixed background, this increase in sociability is consistent with findings from some hybrid CC mouse lines, which exhibit a modest increase in social interaction relative to their inbred B6 parental strain^22^.

Crucially, the enhanced social interaction observed in B6;129 mice cannot be attributed to major differences in olfaction or social memory, as both backgrounds performed almost similarly in those tasks. This is important considering that olfactory processing plays a central role in rodent social interactions^47^ and that olfactory deficits are a known driver of impaired social behavior in ASD mouse models (e.g., BTBR vs. B6)^11^. However, it remains possible that differences in early-life maternal care between strains contributed to adult behavioral outcomes. While some studies report no difference in maternal behavior^48^, others describe shorter pup retrieval latency in 129 females^49^, which may influence offspring social behavior.

At a molecular level, oxytocin receptor levels may help to explain the observed differences in social interaction between the two backgrounds. For example, B6 mice exhibit reduced oxytocin receptor density, particularly in the medial preoptic area (MPOA), compared to 129 mice^48^. Moreover, levels of oxytocin receptors in the nucleus accumbens have been implicated in regulating social bonding in monogamous prairie voles^50^, and oxytocin receptor signaling in this brain region controls social reward and interaction with unfamiliar conspecifics in mice^51^. Oxytocin receptor levels may specifically underlie the background difference in unfamiliar, but not familiar, social interaction that we observed. Supporting this, we have previously demonstrated distinct oxytocin kinetics and social plasticity during these two different types of social interactions in B6;129 mice^42^.

Similar to social interaction, we observed that WT mice on the mixed B6;129 background displayed increased self-grooming behavior, which was not found in inbred B6 and previously reported in 129 lines^52^. This increased repetitive behavior was modest compared to *Shank3* KO mice in the same background^43^. In contrast, only B6 mice showed heightened anxious-like behavior, consistent with previous reports comparing inbred B6 and 129 lines^6,12,48,52^. Although not significant in our study, increased locomotor activity was also reported in B6 mice^6,12,48,52^. No major differences in learning, memory, or cognitive flexibility were observed between the two backgrounds. However, subtle effects on long-term memory or age-related decline have been previously documented^6,23^, and such differences cannot be excluded in our study, which was performed with younger 2-month-old mice.

Notably, social interaction and motor stereotypies, two core behavioral features relevant to neurodevelopmental and psychiatric conditions, particularly ASD, emerged as the most discriminating behavioral parameters between the inbred B6 and mixed B6;129 genetic mouse backgrounds. Despite the expected behavioral variability associated with their genetic background, B6;129 mice consistently amplified sociability and repetitive behaviors, facilitating the potential detection of phenotypes in *Shank3* KO mice^43^. Interestingly, B6;129 mice were also less sensitive to social isolation, as previously reported^42^, compared to the inbred B6 background, suggesting improved behavioral adaptability, along with greater physiological robustness^23^. The genetic phenomenon in which hybrid offspring display traits that exceed those observed in either parental population, is known as transgressive segregation^53,54^. This phenomenon has also been reported for working memory in recombinant BXD mouse strains, compared to the parental lines^55^.

Our findings align with studies on CC and BXD lines, which demonstrated that genetic diversity can either reveal or attenuate behavioral phenotypes of the inbred B6 parental strain^22,56^. The novelty of our study is that a single mixed background provides genetic heterogeneity comparable to that obtained using more than 20 lines in hybrid or outbred lines, thereby reducing ethical concerns (number of animals) and experimental costs. A limitation of this study, however, is that only one mixed background was used. Greater genetic heterogeneity could be achieved using other Diversity outbred (DO) mice^26,27^ or wild-derived mice^21^.

Importantly, our findings address common concerns that mixed backgrounds might mask the effects of genetic manipulations due to increased variability. In fact, both genetic backgrounds (more pronounced in B6 mice) showed enhanced behavioral variability following chronic isolation. Conversely, reliance on a single inbred strain may restrict generalizability. For example, only B6 mice displayed increased social interaction in response to isolation (with an isolated mouse), a pattern not observed in the B6;129 line. This finding indicates strain-specific responses that may limit translational relevance when using inbred models alone^14,15^.

Overall, our study highlights the genetic and behavioral relevance of using the mixed B6;129 background for modeling neurodevelopmental and psychiatric conditions. Compared to the inbred B6 line, the B6;129 background better captures the behavioral heterogeneity characteristic of these conditions in humans. Incorporating genetic diversity into preclinical neurological research not only improves the reproducibility of findings but also enhances their translational value, addressing key limitations of conventional inbred models.

## Methods

### Use of experimental animals

We confirm that all experiments were approved by the local ethical Committee CEEA Val de Loire N°19 and the French ministry of teaching, research and innovation animal care committees (APAFIS #18035-2018121213436249) and adhered to the French and European Directives for the use of live mice. We confirm that all experiments were performed and reported in accordance with the ARRIVE guidelines^57^. Combined O_2_ with CO_2_ was used for mouse euthanasia, a commonly utilized method and accepted norm consistent with the veterinary best euthanasia practice for animal group.

### Animals

Following breeding of male or female 100% pure C57BL/6J (B6) with respective female or male 100% pure 129/S2SvPas (129) (Charles River, France), the hybrid F1 generation was outbred over five generations to generate the 50%-50% mixed C57BL/6J;129/S2SvPas (B6;129) background mice. The mice were housed in the same room of a conventional animal facility under 12-hour light/dark cycle, with food and water *ad libitum*, controlled temperature (21 °C), and humidity (50%), in social groups of 2–4 sex- and genotype-matched animals per type 2 conventional cage or isolated for four weeks as previously done^43^ prior to behavioral phenotyping. All mice used in the experiments were two-month-old sexually naïve males and females at the start of the behavioral experiments from 2 to 4 distinct cohorts.

### Behaviors

All behavioral tests were conducted at the start of the light phase in a dedicated, quiet room, with one assay per day under a dim light intensity of 15 lx (unless stated otherwise) following the experimental timelines (Fig. 1). Standard behavioral equipment and tasks were used to evaluate specific behaviors across multiple assays with all trials recorded and analyzed *a posteriori*, as previously described^42,43^. The experiments were performed by a trained experimenter, mostly female, who was not blinded to the background or housing conditions. Parameters in the open field, Y-maze, elevated plus maze, and habituation in the three-chambered test were automatically analyzed using the animal tracking ANY-maze software (Stoelting, Ireland), while complex social interactions were automatically scored using the Live Mouse Tracker (LMT)^36^. Motor stereotypies, olfaction, latency to feed in the novelty-suppressed feeding, and social interaction tests were manually scored *a posteriori* by a trained experimenter blinded to the conditions using BORIS Software^58^ or directly for the number of buried marbles in the marble-burying assay.

### Sociability and social memory tests

In the reciprocal social interaction test^32,33^, mice interacted with a sex-, background-, and genotype-matched cage mate or an unfamiliar mouse for 10 min in the open field arena (40 × 40 cm)^42^. In the three-chambered test^32,33^, experimental animals were exposed to subsequent habituation (empty vs. empty cylinders located in the opposite chambers), sociability (sex-matched unfamiliar WT mouse 1 (trained twice over 20 min in a cylinder prior to testing) vs. Lego Duplo toy), social novelty (familiar mouse 1 vs. a novel unfamiliar WT trained mouse 2 replacing the toy), and mate preference (familiar mouse 2 vs. a cage mate replacing mouse 1) phases on the same day^42^. Complex social interactions in the 50 × 50 cm LMT arena^36,43^ were examined across distinct and consecutive trials with up to 4 familiar cage mates or a mix of unfamiliar age- and sex-matched B6 and B6;129 mice. In the social recognition habituation-dishabituation memory test adapted from^35^, after 5 min habituation in a conventional cage with clean bedding, experimental mice were subjected to three consecutive 5-min trials with the same unfamiliar age-, sex-matched B6;129 WT mouse, separated by one and 24-hour intervals, followed by a fourth trial with a novel unfamiliar B6;129 WT mouse after one-hour interval. “Nose contact” was defined as oral-oral and oral-genital contacts, unless specified.

### Olfactory habituation/dishabituation test adapted from^34^

After 5 min habituation in a conventional cage with clean bedding and a clean 2 cm x 2 cm Whatman paper tapped to the cage wall at the height of 4 cm (later soaked with 20 µL of each odor and replaced by a clean paper between each trial), sniffing (< 1 cm) of the soaked paper was measured by exposing individual mouse to three 2-min trials of neutral blossom flower odor (diluted 1:2 in saline), followed by three trials of same-sex urine and three trials of opposite-sex urine, with a one-minute inter-trial interval. Urine was collected from ten B6;129 WT males or females, not included in the study.

### Compulsive, repetitive, and stereotyped behaviors

Repetitive behavior (self-grooming and digging) and motor stereotypies (vertical jump, circling, head shakes, and scratching)^33^, as well as immobility and rearing (spatial exploration), were measured for each mouse placed in a clear and clean conventional cage filled with a 3–4 cm thick layer of fresh litter for 10 min at 400 lx. Compulsive/anxious-like behavior was assessed in the marble-burying test^33^ by measuring the number of marbles buried more than two-thirds by each mouse exposed to 20 glass marbles (1.5 cm diameter) equally spaced on 4 cm thick fresh litter in a clean conventional cage closed with a transparent plexiglass for 15 min. To assess perseverative behavior, the percentage of spontaneous alternation (SPA), alternative arm returns (AAR), and same arm returns (SAR; perseverative behavior) was determined from a triplet of arm entries for each mouse placed in a (35 × 5 × 10 cm) spatial Y-maze test^33^ for 5 min.

### Locomotion and anxious-like behavior

Locomotion (total distance traveled and mean speed) and time in the center were evaluated for each mouse in the open field arena for 10 min^39^. In the novelty-suppressed feeding test^40^, the latency to reach three pellets of standard lab chow placed on a white tissue in the center of the open field arena filled with a thin layer of fresh litter, was measured for each 16 h-food-deprived mouse exposed to 60 lx. Higher latency is considered a proxy for anxious-like behaviors. In the elevated plus maze test (4 arms, 5 cm wide and located 80 cm above the ground)^39^, the number and time spent in the opened or closed arms, the anxiety index measured as the time spent in the closed arm / total time in both arms, as well as distance traveled and immobility for each mouse, were evaluated for 5 min.

### Spatial location and novel object recognition tests adapted from^59^

In the spatial object location recognition test, after two habituations for 10 min with two Lego Duplo toys located on the same side of the center border in the open field area with 1.5 h intervals, spatial memory of the mice was tested for 10 min by placing one Duplo (“spatial object”) to the diagonal side of the arena while the other one remained at the same position (“old object”). In the novel object recognition test, after the same two habituations, mice were tested for 10 min 24 h later for mid-term object recognition memory by replacing one toy by a bigger Lego Duplo toy with a different color (“novel” object) and, after 1.5 h interval for short-term recognition memory by replacing the small habituation Duplo by a Falcon tube (second “novel” object). The discrimination index was calculated as the time spent with the spatial or novel object – old object / total time with the objects.

### Experimental design and statistical analysis

Power analysis of animal sample size calculated a minimum of 8 mice per group (power of 80%, alpha level of 5% (*p* = 0.05), differences in means of 41%). The sample size was greater than 8 animals per group, as all animals within a cage were tested to avoid any bias due to dominance/subordinate effect and potential death of animals. The exact sample sizes and sex ratio are provided in Tables S1-3.

Criteria to exclude animals from behavioral tests were over 30% of the time spent immobile and/or a minimum of two explorations of all arms or compartments. The animal outliers (± 3 standard deviations) detected in each behavioral assay were removed. Therefore, a total of 14 animals (11 B6;129 and 3 B6 mice) were excluded from the analysis. All data and statistical analyses were performed using R software (version 4.3.1). For comparisons between groups, the non-parametric Kruskal-Wallis tests with Dunn’s posthoc tests were performed using the rstatix package^60^. P-values were adjusted with Benjamini-Hochberg correction^61^. The correlation of social interaction parameters from the reciprocal social interaction test and the sociability phase of the three-chambered test was assessed using the ggpmisc package^62^. Principal component analysis (PCA) was performed using the FactoMineR package^63^ to visualize the inter-individual variability in the reciprocal social interaction, three-chambered, motor stereotypies, Y-maze, open field, and novelty-suppressed feeding tests in each background. Missing values (NAs) in the behavioral dataset used in PCA were imputed by replacing each missing entry with the mean value of the corresponding variable across all samples. This was done using a column-wise operation in which, for each variable, all missing values were substituted by the variable’s calculated mean, excluding NAs. Sex effects were tested as a variable, except in Fig. 6, where number of animals (*N* < 8) was too low to assess sex effect. To assess whether behavioral inter-individual variability in principal components differed between backgrounds, individual scores for each principal component were compared between backgrounds by one-way ANOVA, followed by Tukey’s posthoc test using the car package^64^. To reveal the most discriminating behavioral parameters contributing to the behavioral inter-individual variability in the PCA, a biplot of individuals and the five most discriminant variables was realized using the factoextra package^65^. Variance in behavioral parameters between backgrounds was compared by Levene’s test using the car package^64^. Mean ± standard deviation (sd), and statistics, along with sex effects, are reported in Tables S1-S3.

## Supplementary Information

Below is the link to the electronic supplementary material.

Supplementary Material 1

Supplementary Material 2

Supplementary Material 3

Supplementary Material 4

## Data Availability

Mean ± standard deviation, and statistics are represented in **Tables S1–3** . The dataset and R codes supporting the conclusions of this article is available in the Zenodo repository, https://zenodo.org/records/16354202.

## Abbreviations

ASD: Autism spectrum disorder
CC: Collaborative Cross
DO: Diversity outbred
KO: Knockout
LMT: Live Mouse Tracker
PCA: Principal component analysis
Sd: Standard deviation
WT: Wildtype
