# Supplementary material for: Mixed genetic background better recapitulates developmental and psychiatric phenotypes and heterogeneity than inbred C57BL/6J mice
Source: Sci Rep. 2025 Nov 7;15:39130. doi: 10.1038/s41598-025-26215-6 (PMC12595115; doi:10.1038/s41598-025-26215-6)
Supplement: Supplementary file 4 — Supplementary Material 4 [file 41598_2025_26215_MOESM4_ESM.docx]

**SUPPLEMENTAL INFORMATION**

**SUPPLEMENTAL FIGURES**

**Supplemental figure 1. Enhanced sociability with unfamiliar mice in mixed B6;129 mice is present in both sexes.** In the 10-min phases of the three-chambered test, both male and female B6;129 mice (orange) displayed enhanced (unfamiliar WT mouse 1 *vs* object; **A**), social novelty preference (familiar WT mouse 1 *vs* novel unfamiliar WT mouse 2; **B**) and familiar cage mate (familiar cage mates *vs* familiar WT mouse 2; **C**) preference compared to corresponding sexes of B6 mice (green). In the 10-min reciprocal social interaction assay no difference was observed between sexes in either B6 or B6;129 backgrounds in interaction with familiar cage mates (**D**), while both male and female B6;129 mice displayed enhanced social interaction with a background-, sex- and age-matched unfamiliar mouse compared to sex-matched B6 mice (**E**). In the 5-minute social memory test (**F**), no difference in social recognition and habituation to the WT interactors was observed between sexes in both B6 and B6;129 backgrounds across the four trials. In the odor habituation/dishabituation assay (**G**), B6;129 female mice displayed reduced interest in the opposite sex WT urine (O3) compared to B6;129 males, while no sex difference was observed in the B6 background. Data are presented as individual data, mean ± sd (statistics, n, and sex ratio in **Table S1**). Groups were compared by Kruskal-Wallis tests followed by Dunn post hoc tests, with stars indicating background effect, hash sex effect, and dollar signs chamber effect (P = adjusted p-value). *, # or $ p < 0.05; **, ## or $$ p < 0.01; ***, ### or $$$ p < 0.001; ****, #### or $$$$ p < 0.0001.

**Supplemental figure 2. No sex differences in social exploration and following in the Live Mouse Tracker.** In the 10-minute complex social interaction in the Live Mouse Tracker with either cage mates (**A**) or a mix of 2 unfamiliar B6 mice and 2 unfamiliar B6;129 mice (**B**) no sex difference was observed in the time spent in oral-genital contact or time spent following in either B6 (green) or B6;129 background (orange). Data are presented as individual data, mean ± sd (statistics, n and sex ratio in **Table S2**). Groups were compared by Kruskal-Wallis tests followed by Dunn post hoc tests, with stars indicating background effect and hash sex effect (P = adjusted p-value. * or # p < 0.05; ** or ## p < 0.01; *** or ### p < 0.001.

**Supplemental figure 3. Males from the mixed B6;129 background mice display increased self-grooming and digging behaviors.** In the 10-minute motor stereotypies (**A**), male B6;129 mice (orange) displayed reduced number and time spent digging, and increased time spent self-grooming compared to B6 males (green), and no difference in the head shake episodes, while no difference was observed between the female B6 and B6;129 mice. No sex difference in the marble burying test (**B**) or in the Y maze alternation pattern (**C**) was observed between B6 mice and B6;129 mice. Data are presented as individual data, mean ± sd (statistics, n and sex ratio in **Table S1**). Groups were compared by Kruskal-Wallis tests followed by Dunn post hoc tests, with stars indicating background effect, and hash sex effect (P = adjusted p-value). * or # p < 0.05; ** or ## p < 0.01; *** or ### p < 0.001.

**Supplemental figure 4. Anxious-like behavior in the B6 background is restricted to females.** No sex difference was observed in the spatial object location (**A**), novel object recognition (**B**) memory tests, or in the traveled distance in the open field (**C**), in which male and female B6 and B6;129 mice performed similarly. No sex-specific difference was observed in the elevated plus maze (**D**) or time in the center in the open field arena (**E**), while increased anxious-like behavior in the B6 background in the novelty-suppressed feeding test was restricted to female mice (**F**). Data are presented as individual data, mean ± sd (statistics, n, and sex ratio in **Table S1**). Groups were compared by Kruskal-Wallis tests followed by Dunn post hoc tests, with stars indicating background effect and hash sex effect (P = adjusted p-value). * or # p < 0.05; ** or ## p < 0.01; *** or ### p < 0.001.

**Supplemental figure 5. Higher variability in the B6;129 background is present in both sexes.** The increased variability in B6;129 background (orange) compared to B6 (green), as evidenced by the principal component analysis based on the behavioral phenotypes in the reciprocal social interaction, three-chambered, motor stereotypies, Y-maze, open field, and novelty-suppressed feeding tests (left), was present in both sexes (**A**), confirmed by a significant increase of the scores for the first principal component in both male (diamond shapes) and female (circle shapes) B6;129 mice compared to B6 (right). The higher variability in the B6;129 background observed in the principal component analysis (left), based on the behavioral phenotypes in the Live Mouse Tracker (**B**), was present in both male and female mice, confirmed by an increase in the scores for the second component for both male and female B6;129 mice compared to B6 counterparts (right). Data are presented as individual data, mean ± sd (statistics, n, and sex ratio in **Table S1**). Groups were compared by Kruskal-Wallis tests followed by Dunn post hoc tests, with stars indicating background effect and hash sex effect (P = adjusted p-value). * or # p < 0.05; ** or ## p < 0.01; *** or ### p < 0.001.

**SUPPLEMENTAL TABLES**

**Table S1. Mean and statistics from standardized behavioral tests performed in adult mice.**

**Table S2. Mean and statistics from the social interaction in the Live Mouse Tracker.**

**Table S3. Mean and statistics from isolated and WT mice.**
